# Imipramine blocks acute silicosis in a mouse model

**DOI:** 10.1186/s12989-017-0217-1

**Published:** 2017-09-11

**Authors:** Rupa Biswas, Kevin L. Trout, Forrest Jessop, Jack R. Harkema, Andrij Holian

**Affiliations:** 1 Center for Environmental Health Sciences, Department of Biomedical and Pharmaceutical Sciences, University of Montana, Missoula, MT 59812 USA; 2 Department of Pathobiology and Diagnostic Investigation, Michigan State University, East Lansing, MI 48824 USA

**Keywords:** Silica, Silicosis, Particles, Imipramine, Macrophage, Lysosome, Inflammation, Toxicology

## Abstract

**Background:**

Inhalation of crystalline silica is associated with pulmonary inflammation and silicosis. Although silicosis remains a prevalent health problem throughout the world, effective treatment choices are limited. Imipramine (IMP) is a FDA approved tricyclic antidepressant drug with lysosomotropic characteristics. The aim of this study was to evaluate the potential for IMP to reduce silicosis and block phagolysosome membrane permeabilization.

**Methods:**

C57BL/6 alveolar macrophages (AM) exposed to crystalline silica ± IMP in vitro were assessed for IL-1β release, cytotoxicity, particle uptake, lysosomal stability, and acid sphingomyelinase activity. Short term (24 h) in vivo studies in mice instilled with silica (± IMP) evaluated inflammation and cytokine release, in addition to cytokine release from ex vivo cultured AM. Long term (six to ten weeks) in vivo studies in mice instilled with silica (± IMP) evaluated histopathology, lung damage, and hydroxyproline content as an indicator of collagen accumulation.

**Results:**

IMP significantly attenuated silica-induced cytotoxicity and release of mature IL-1β from AM in vitro. IMP treatment in vivo reduced silica-induced inflammation in a short-term model. Furthermore, IMP was effective in blocking silica-induced lung damage and collagen deposition in a long-term model. The mechanism by which IMP reduces inflammation was explored by assessing cellular processes such as particle uptake and acid sphingomyelinase activity.

**Conclusions:**

Taken together, IMP was anti-inflammatory against silica exposure in vitro and in vivo. The results were consistent with IMP blocking silica-induced phagolysosomal lysis, thereby preventing cell death and IL-1β release. Thus, IMP could be therapeutic for silica-induced inflammation and subsequent disease progression as well as other diseases involving phagolysosomal lysis.

**Electronic supplementary material:**

The online version of this article (10.1186/s12989-017-0217-1) contains supplementary material, which is available to authorized users.

## Background

Silicosis is a lung disease caused by inhalation of crystalline silica and is most prevalent as a result of occupational exposures [1, 2]. The seriousness of the disease is reflected by the morbidity and disabling illnesses that continue to occur among workers [3]. Silicosis can be categorized into subtypes based on duration of exposure and associated pathology. In a general sense, chronic silicosis develops after at least 10 years of low concentration exposures, accelerated silicosis develops after 5-10 years of medium concentration exposures, and acute silicosis develops after a few weeks to 5 years of high concentration exposures [4]. Silicoproteinosis is a common distinguishing pathology of acute silicosis, and these terms are often used interchangeably. Most forms of human silicosis are difficult to mimic experimentally due to the long latency period for disease development, but animal models of silica exposure have been shown to produce similar characteristics in a more rapid timeline [5–7]. Currently, treatment choices for silicosis are limited and accompanied by complex adverse outcomes [4]. Therefore, it remains important to understand the mechanism of silica-induced inflammation and disease progression in order to develop effective therapeutics.

When respirable particulates of 2.5 μm or less deposit in the pulmonary alveolus, they are cleared by alveolar macrophages (AM) and are predominantly contained in phagosomes [8, 9]. Lysosomes fuse with the phagosomes to form phagolysosomes, in which lysosomal enzymes attempt to degrade phagocytosed particulates. However, a number of particulates including silica cannot be degraded, which may contribute to phagolysosomal membrane permeabilization (LMP) [10, 11]. Nevertheless, the exact cause of particle-induced phagolysosomal instability is not clearly understood.

LMP results in the release of lysosomal proteases to the cytoplasm that have been reported to contribute to cytotoxicity [12–16] and to trigger assembly of the multiprotein complex, NLRP3 inflammasome [17]. The function and regulation of inflammasomes has been reviewed elsewhere [18–22]. NLRP3 inflammasome assembly leads to activation of Caspase-1, which cleaves pro-Interleukin (IL)-1β and pro-IL-18 to their active forms. Another signal is required to activate the NF-κB pathway, such as lipopolysaccharide (LPS), IL-1β, high mobility group box 1 (HMGB1) protein [23], or other damage/pathogen-associated molecular patterns (DAMP/PAMP). NF-κB activation upregulates transcription of inflammasome components, pro-IL-1β, and pro-IL-18. The release of mature IL-1β and IL-18 have been closely associated with acute inflammation and development of lung fibrosis [24, 25]. It should be noted that although inflammasome activation is typically considered to be the primary mechanism for IL-1β processing, it has been suggested that there may also be smaller contributions from inflammasome-independent mechanisms in vivo [20].

Since LMP plays an important regulatory role in NLRP3 inflammasome activation and subsequent inflammation, blocking LMP would be a target for novel therapeutics for inflammatory diseases linked to lysosomal instability. For example, a drug or an agent that can stabilize the lysosome and prevent membrane permeabilization may have the potential to attenuate downstream inflammation. A potential anti-LMP agent is imipramine (IMP), a U.S Food and Drug Administration approved tricyclic antidepressant drug with lysosomotropic properties [26]. Pretreatment with IMP has been reported to attenuate the inflammatory response and improve survival in an LPS-induced acute lung injury model [27]. In another study, the authors reported that IMP and surfactant synergistically suppressed acid sphingomyelinase (aSMase) activity in lung tissue, reduced ceramide generation, and improved pulmonary function in a newborn piglet lavage model [28]. However, IMP has not been examined in models of particulate-induced inflammation. Therefore, the potential of IMP to inhibit inflammation following silica exposure was examined in this study.

We hypothesized that IMP will stabilize lysosomes, thereby decreasing NLRP3 inflammasome activation and downstream inflammation. In vitro studies were conducted using AM from C57Bl/6 mice to evaluate the effect of IMP on IL-1β release and cytotoxicity. In vivo studies with C57Bl/6 mice were conducted to determine if IMP could decrease silica-induced acute inflammation and pathology. Furthermore, we determined the potential of IMP as a protective and a therapeutic agent in a long-term crystalline silica exposure model.

## Results

### Imipramine inhibited silica-induced IL-1β release and cytotoxicity in vitro

**Fig. 1:**
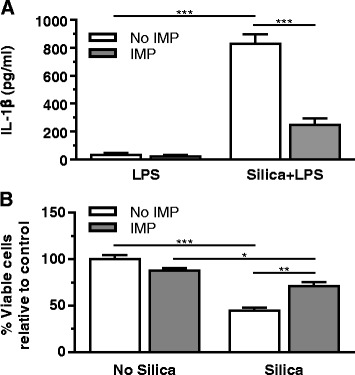
In vitro IL-1β release and cytotoxicity in AM treated with silica ± imipramine. AM treatment conditions include IMP for 30 min, followed by exposure to LPS and silica for 24 h. **a** IL-1β release was measured by ELISA. **b** MTS assay results are expressed as cell viability relative to no silica, no IMP control. *n* ≥ 3 mice per treatment condition

Primary AM were used to evaluate the effects of IMP on IL-1β release and cytotoxicity. Isolated AM were treated ± IMP (25 μM) for 30 min and then exposed to LPS (20 ng/ml) ± silica (100 μg/ml). The results in Fig. 1a show silica leads to a significant increase in IL-1β release compared to cells treated with LPS only. IMP pretreatment significantly inhibited IL-1β release in silica-exposed AM. Similar results were obtained using cell line THP-1 (Additional file 1: Figure S1), which is a human monocytic leukemia line that is commonly used for particle toxicology studies in our laboratory and others [29, 30]. These results demonstrate that IMP can significantly block silica-induced IL-1β release in vitro.

AM cytotoxicity was examined for two purposes: (1) to determine whether the observed decrease in IL-1β was due to any unexpected toxicity of IMP, and (2) to evaluate the effect of IMP on silica-induced cytotoxicity. Isolated AM were pretreated ± IMP for 30 min then exposed to silica overnight. AM exposed to silica showed the expected significant loss in cell viability compared to baseline control (Fig. 1b). Silica-induced cytotoxicity was significantly lowered by IMP pretreatment. Taken together, IMP blocked both the silica-induced IL-1β release and cytotoxicity.

### Imipramine pre-treatment reduced short-term silica-induced inflammation in vivo

**Fig. 2:**
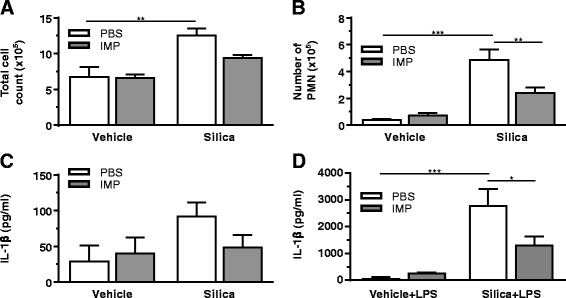
IMP pre-treatment effect on acute inflammation induced by silica. Mice were treated with IMP or PBS for 30 min, then exposed to silica or vehicle. Whole lung lavage was collected after 24 h. **a** Total number of cells in the lavage fluid. **b** Polymorphonuclear (PMN) leukocytes in lavage were assessed by staining. **c** IL-1β levels in lavage fluid. **d** IL-1β in supernatant of isolated AM exposed to LPS ex vivo for an additional 24 h. *n* ≥ 3 mice per treatment condition

Since IMP was effective in decreasing silica-induced IL-1β release from macrophages in vitro, it suggested that IMP could be anti-inflammatory in vivo. Therefore, mice were given IMP (25 mg/kg) or vehicle (100 μl) by intraperitoneal (IP) injection 30 min before silica (1 mg/mouse in 25 μl) or vehicle (PBS (sham); 25 μl) exposure. After 24 h, whole lung lavage was analyzed by cell differentials and ELISA for IL-1β. IMP pretreatment did not significantly change the total cell counts (Fig. 2a) and the number of eosinophils and lymphocytes were negligible. In the silica-exposed group there was a significant increase in neutrophils in lavage fluid, and IMP pretreatment significantly inhibited neutrophil infiltration following silica exposure (Fig. 2b). Furthermore, IL-1β levels in lavage fluid were assessed (Fig. 2c). Trends suggest IL-1β is increased by silica, which is reduced by IMP treatment. Though the inhibition of IL-1β release into the lavage fluid did not achieve statistical significance, it followed a similar pattern as observed in vitro.

In order to determine whether IMP had specifically blocked IL-1β production by macrophages in vivo, AM were collected from the lavage fluid in the 24 h experiment above. The lavaged cells were then cultured ex vivo for 24 h ± LPS (20 ng/ml). There was a significant increase in IL-1β release in the silica-exposed group compared to that in the controls, which was significantly reduced by IMP pretreatment (Fig. 2d). Therefore, IMP given IP was sufficient to target AM and block silica-induced release of IL-1β. Overall, the results indicate that the anti-inflammatory effect of IMP following silica exposure followed the same pattern in vitro, in vivo and ex vivo.

### Imipramine post-treatment reduced short-term silica-induced inflammation in vivo

**Fig. 3:**
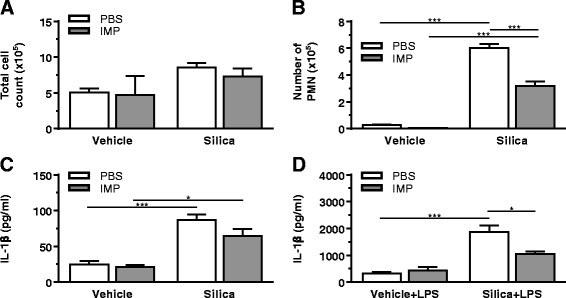
IMP post-treatment effect on acute inflammation induced by silica. Mice were exposed to silica or vehicle for 30 min, then treated with IMP or PBS. Whole lung lavage was collected after 24 h. **a** Total number of cells in the lavage fluid. **b** Polymorphonuclear (PMN) leukocytes in lavage were assessed by staining. **c** IL-1β levels in lavage fluid. **d** IL-1β in supernatant of isolated AM exposed to LPS ex vivo for an additional 24 h. *n* ≥ 3 mice per treatment condition

In the studies above, IMP was given to mice prior to administration of silica. In order to determine whether IMP would also be effective if given after onset of silica-induced inflammation, mice were first exposed to silica (1 mg/mouse) or vehicle (25 μl). After 30 min, mice were given IMP (25 mg/kg in PBS) or PBS alone by IP injection. After 24 h, whole lung lavage was analyzed by cell differentials and ELISA for IL-1β. IMP post-treatment did not significantly change the total cell counts (Fig. 3a) and the number of eosinophils and lymphocytes were negligible. Silica leads to significant neutrophil influx, which is reduced by IMP treatment (Fig. 3b). IL-1β was significantly higher in the group exposed to silica compared to the vehicle control (Fig. 3c). In order to determine whether IMP was acting on AM specifically in this model, the lavaged cells were cultured ex vivo for 24 h in the presence and absence of LPS (20 ng/ml). There was a significant increase in IL-1β release in the silica-exposed group compared to control (Fig. 3d). IMP pretreatment in vivo significantly decreased IL-1β release from AM following silica exposure. Similar trends are observed regardless of whether IMP is given before (Fig. 2) or after (Fig. 3) silica.

### Imipramine concomitant treatment reduced long-term silica-induced pathology in vivo

The data thus far demonstrated that IMP was effective in blocking acute inflammation caused by silica exposure. Because fibrosis is a result of chronic inflammation, additional studies were completed to determine whether continuous treatment with IMP could block the longer-term effects of silica exposure. Therefore, mice were given IMP in osmotic pump implants to continuously release IMP (or PBS sham) as described in Methods. Simultaneously, the mice were given 1 mg silica and three additional doses of 1 mg silica were given 7, 14 and 21 days later to simulate an extended exposure as previously described [31, 32]. At the end of six weeks, the lungs were examined by histology and quantification of collagen content, a hallmark of lung fibrosis.

**Fig. 4:**
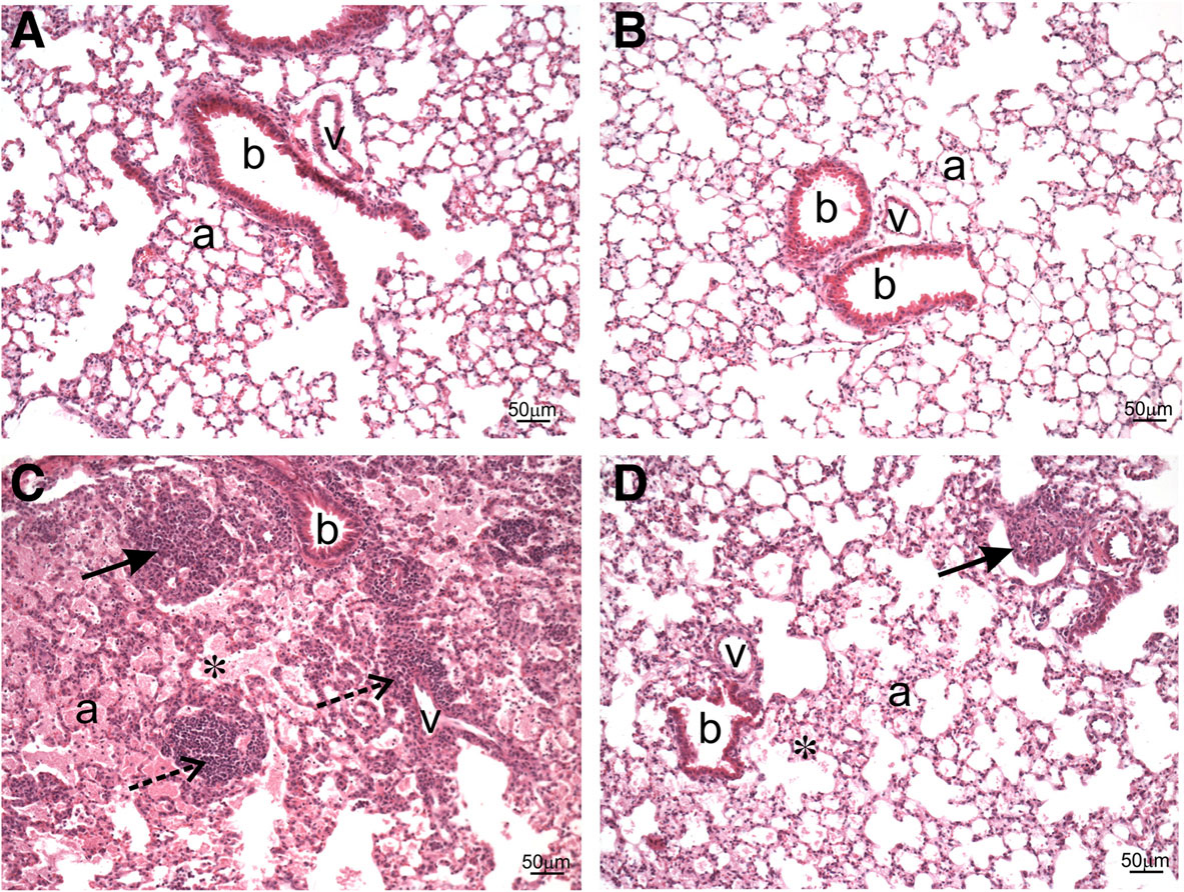
Histopathology from IMP co-administration study. Mice were exposed to silica or vehicle weekly for the first four weeks, while receiving IMP or PBS by osmotic pump for entire six week duration. Light photomicrographs are H&E-stained lung tissue sections from mice treated with (**a**) vehicle, (**b**) IMP, (**c**) silica, and (**d**) silica + IMP. Silica-induced histopathology included alveolar proteinosis (asterisk), microgranuloma (solid arrow), perivascular and peribronchioloar ectopic lymphoid tissue (dashed arrow; lymphoplasmacytic infiltrate). IMP treatment reduced severity of silica-related lesions, with minimal alveolar proteinosis and a small microgranuloma. Abbreviations: a = alveolar parenchyma; b = bronchiolar airway; v = blood vessel. Tissue was observed from *n* ≥ 3 mice per treatment condition

**Fig. 5:**
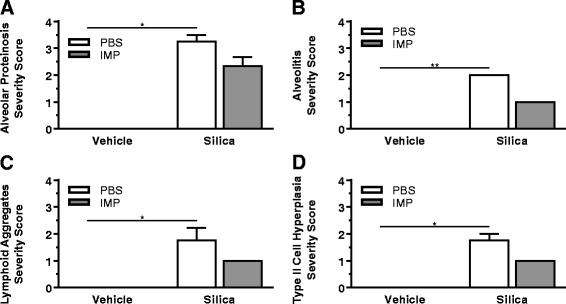
Semi-quantitative analysis of histopathology. Mice were exposed to silica or vehicle weekly for the first four weeks, while receiving IMP or PBS by osmotic pump for entire six week duration. Multiparametric scoring of H&E-stained lung tissue was completed using an ordinal scale ranging from no significant pathology (score: 0) to severe pathology (score: 5). **a** Alveolar proteinosis. **b** Alveolitis including mixed inflammatory cell infiltrates, microgranulomas, and associated interstitial fibrosis. **c** Perivascular/peribronchiolar lymphoid aggregates. **d** Type II alveolar epithelial cell hyperplasia. Tissue was scored from n ≥ 3 mice per treatment condition

A board-certified veterinary pathologist with expertise in respiratory pathobiology of inhaled toxicants in laboratory rodents examined the prepared lung tissue sections by light microscopy. Tissues were examined from at least three mice per treatment group. The pathologist had no knowledge of the exposure history of the individual mice prior to his assessment of pulmonary histopathology. No lung lesions were found in mice that received vehicle alone, regardless of IMP treatment. In contrast, mice instilled with silica without IMP treatment had conspicuous lung lesions consisting of widespread alveolar proteinosis associated with cellular debris and a mixed inflammatory cell influx of neutrophils and mononuclear cells (mainly macrophages/monocytes and lesser numbers of lymphocytes), focal microgranulomas (mainly epithelioid cells and macrophages with lesser numbers of neutrophils), perivascular and peribronchiolar ectopic lymphoid tissue (lymphoplasmacytic), and alveolar type II cell hyperplasia in the affected areas of the lung parenchyma (Figs. 4 and 5).

**Fig. 6:**
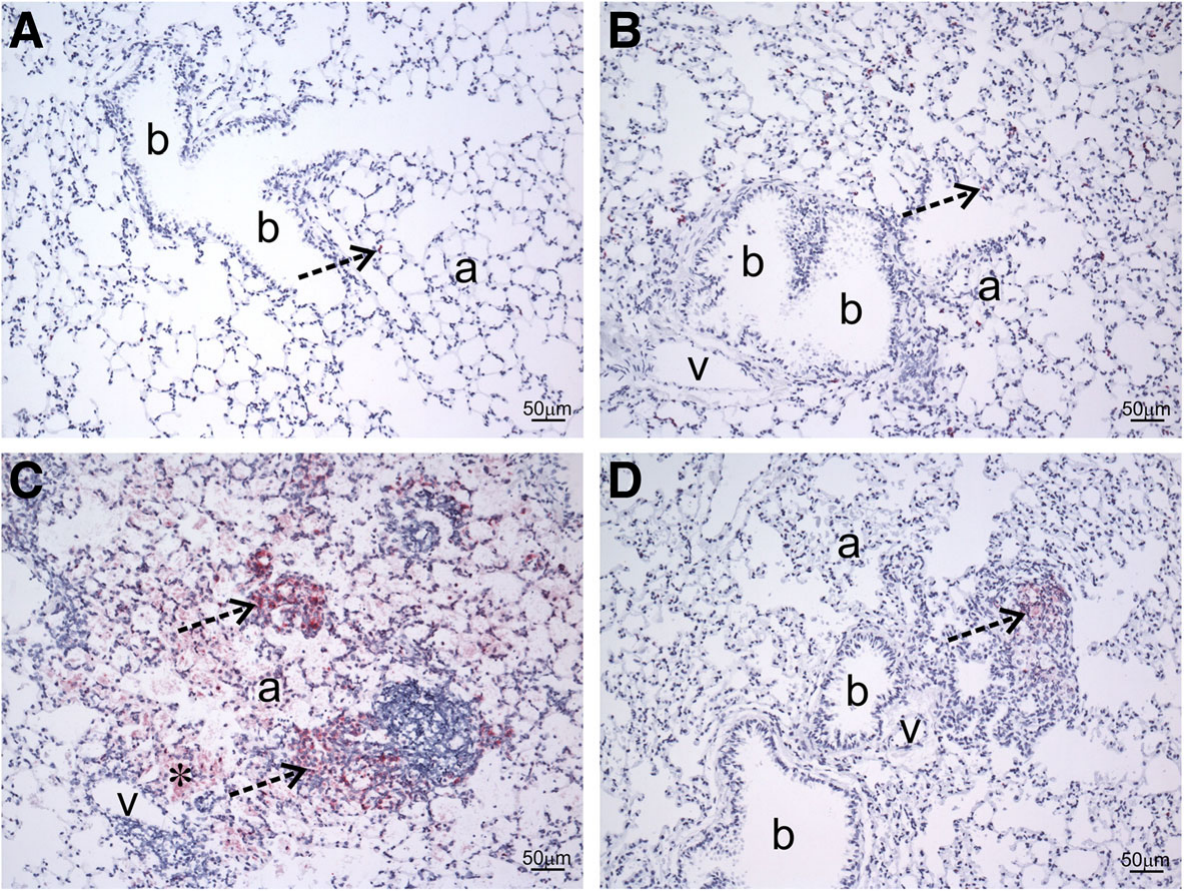
Immunohistochemistry from IMP co-administration study. Mice were exposed to silica or vehicle weekly for the first four weeks, while receiving IMP or PBS by osmotic pump for entire six week duration. Light photomicrographs are lung sections immunohistochemically stained for neutrophils or inflammatory mononuclear cells (red chromogen; dashed arrows) with hematoxylin counterstain from mice treated with (**a**) vehicle, (**b**) IMP, (**c**) silica, and (**d**) silica + IMP. Mice treated with vehicle- or IMP-only had no areas of pulmonary inflammation, with only a few widely scatter neutrophils in the alveolar septa. Silica treatment resulted in conspicuous aggregates of inflammatory monocytes and neutrophils that are often associated with microgranulomas or adjacent to ectopic lymphoid tissue, as well as alveolar proteinosis (asterisk). IMP reduced this silica-associated pathology to only a small solitary aggregate of inflammatory cells. Abbreviations: a = alveolar parenchyma; b = bronchiolar airway; v = blood vessel. Tissue was observed from n ≥ 3 mice per treatment condition

**Fig. 7:**
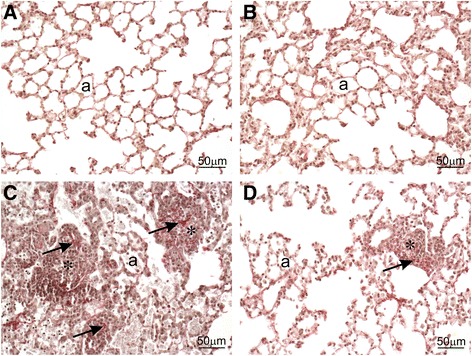
Collagen staining from IMP co-administration study. Mice were exposed to silica or vehicle weekly for the first four weeks, while receiving IMP or PBS by osmotic pump for entire six week duration. Light photomicrographs are lung sections stained for increased collagen (red chromagen; arrows) with hematoxylin counterstain from mice treated with (**a**) vehicle, (**b**) IMP, (**c**) silica, and (**d**) silica + IMP. Silica treatment resulted in a mild increase in collagen associated with microgranulomas (asterisks), which was reduced by IMP. Abbreviation: a = alveolar parenchyma. Tissue was observed from n ≥ 3 mice per treatment condition

Immunohistochemically, there were numerous 7/4 antibody-positive inflammatory cells (neutrophils and inflammatory macrophages/monocytes) in mice treated with silica without IMP (Fig. 6). Also, more picrosirius red-positive staining of collagen was found in the lungs of silica-instilled mice as compared to mice that were not instilled with silica (Fig. 7). This greater staining of lung tissue collagen was most associated with the microgranuloma formation in the alveolar parenchyma.

Interestingly, IMP treatment resulted in less severe silica-associated lesions characterized by mild, widely scattered alveolar proteinosis, minimal ectopic lymphoid tissue around blood vessels or conducting airways, and only a few widely scattered microgranulomas with some 7/4 antibody-positive inflammatory cells and modest picrosirius red-positive collagen (Figs. 4d, 5, 6d, and 7d).

**Fig. 8:**
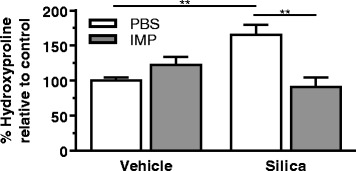
Protective effect of IMP on collagen accumulation during co-administration study. Mice were exposed to silica or vehicle weekly for the first four weeks, while receiving IMP or PBS by osmotic pump for entire six week duration. Hydroxyproline in lungs was measured as an indicator of collagen accumulation. n ≥ 3 mice per treatment condition

Collagen in the lung was measured by hydroxyproline content (Fig. 8). Hydroxyproline content was significantly higher in the silica-exposed group compared to the vehicle-exposed group. IMP treatment significantly attenuated collagen levels in the silica co-administered group. These results demonstrate that the collagen deposition due to extended silica exposure can be attenuated by IMP co-treatment.

### Imipramine post-treatment reduced long-term silica-induced lung injury and collagen deposition in vivo

**Fig. 9:**
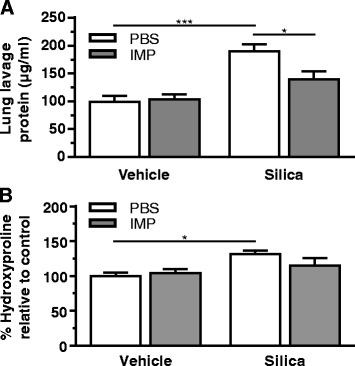
Therapeutic effect of IMP on lung injury and collagen accumulation during post-administration ten-week study. Mice were exposed to silica or vehicle weekly for the first four weeks, then received IMP or PBS by osmotic pump for remaining six weeks. **a** Total protein levels in lavage fluid as an indicator of lung injury. **b** Hydroxyproline in lungs as an indicator of collagen accumulation. n ≥ 3 mice per treatment condition

The studies above demonstrated that IMP reduced pathology and collagen deposition when given at the same time as silica in a long-term silica exposure mouse model. However, this did not establish the potential of IMP to act as a therapeutic to reverse lung injury and collagen accumulation after disease development. In order to test this potential, disease was first induced by administration of silica for four weeks (as described above), then IMP (or sham) was given to mice for six weeks using osmotic pumps. Protein levels in the lavage fluid, a marker of alveolar-capillary barrier damage, were measured as an indicator of lung injury (Fig. 9a). Lung lavage protein levels were significantly increased in the silica and sham-treated group compared to the controls. In the silica and IMP-treated group, lung lavage protein levels were significantly lower compared to the silica and sham-treated group. This result demonstrates that IMP treatment reverses silica-induced lung injury.

Hydroxyproline content was higher in the silica and sham-treated group compared to the controls (Fig. 9b). This high hydroxyproline is an indicator of collagen deposition. Mice exposed to silica no longer had significantly increased collagen deposition when they received IMP as a therapy. Taken together, the long-term in vivo results indicated that IMP treatment, after four weeks of silica exposure, did reverse the development of silica-induced lung pathology.

### Imipramine did not affect silica uptake by macrophages

**Fig. 10:**
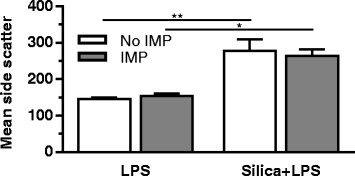
Silica uptake by AM in vitro. AM treatment conditions include IMP for 30 min, followed by exposure to silica for 90 min. Mean side scatter is expressed in arbitrary units. n ≥ 3 mice per treatment condition

The above results demonstrate the anti-inflammatory properties of IMP in silica-induced pathology. A potential explanation for this mechanism of action is that IMP inhibits silica uptake, thereby reducing the amount of silica in phagosomes and decreasing LMP. AM silica uptake was investigated by measuring the increase in side scatter using flow cytometry (Fig. 10). AM were pretreated ± IMP for 30 min and then exposed to silica for 90 min before measurement of side scatter. IMP did not affect AM silica uptake. Also, IMP did not affect phagosome maturation into an acidified phagolysosome (Additional file 1: Figure S2). Therefore, the observed IMP inhibition of inflammation and cytotoxicity was not due to an alteration of the silica uptake mechanism.

### Imipramine inhibits acid sphingomyelinase (aSMase)

**Fig. 11:**
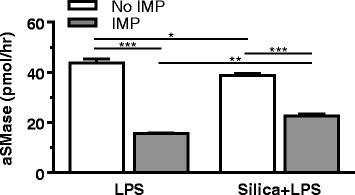
Acid sphingomyelinase (aSMase) in AM in vitro. AM treatment conditions indicated here include IMP for 30 min, followed by exposure to LPS and silica for 2 h. The aSMase activity in cell lysates is expressed as pmol/h. n ≥ 3 mice per treatment condition

IMP treatment reduces phagolysosome membrane permeabilization caused by silica (Additional file 1: Figure S3). A proposed mechanism by which this occurs is through inhibition of lysosomal acid sphingomyelinase (aSMase) by IMP, leading to a change in lipid balance and stabilization of the phagolysosomal membrane. aSMase catalyzes the hydrolysis of sphingomyelin into ceramide at an acidic pH in lysosomes [33, 34]. aSMase activity was measured in AM as described in Methods. IMP significantly inhibited aSMase activity (Fig. 11), which is consistent with the literature [27, 28, 35, 36].

## Discussion

Silica exposure leads to inflammation and fibrosis. Currently, there is no known therapy to reduce this pathology. Following phagocytosis by alveolar macrophages (AM), silica is contained in the phagosome, which subsequently fuses with lysosomes. Crystalline silica leads to phagolysosome membrane permeabilization (LMP) accompanied by release of a lysosomal cathepsins and other hydrolases into the cytoplasm, which consequently leads to NLRP3 inflammasome assembly and Caspase-1 activation [17, 37]. Furthermore, LMP contributes to silica-induced cell death [12, 16]. Therefore, blocking LMP is a good candidate therapeutic target because its significant role in the onset of inflammation [11].

The focus of this study was to determine the potential of imipramine (IMP) to block LMP and inhibit the pathology following crystalline silica exposure. IMP is a lipophilic weak base that crosses the lysosomal membrane and becomes protonated within the lysosome, resulting in the drug getting trapped inside the acidic environment of the lysosome [26]. We hypothesized that IMP stabilization of the lysosome prevents silica-induced LMP, blocking consequent inflammasome activation and downstream pathology.

IMP was effective in blocking silica-induced IL-1β release and cytotoxicity of AM in vitro. Short-term in vivo studies demonstrated that IMP pretreatment resulted in significantly lower silica-induced neutrophilia and a trend indicating lower IL-1β levels. The target of IMP action was confirmed to be macrophages because AM cultured ex vivo produced significantly lower levels of IL-1β. The results from IMP pre-treatment were similar to post-treatment results, when IMP was administered after silica exposure. It was evident by these short-term studies that the anti-inflammatory effect of IMP occurs rapidly and continues for at least 24 h.

Treatment for silicosis should aim at preventing the progression of the disease, reducing inflammation, decreasing fibrosis, and improving quality of life. Repeated administration of silica over a four-week time period in a murine model has previously been shown to result in pathology similar to silicosis in humans [31, 32]. Therefore, the present study aimed to determine whether IMP has the potential to be both a protective and a therapeutic agent following long-term exposure to silica in a murine model. IMP co-administration with silica attenuated acute silicosis pathology and collagen deposition. Similarly, IMP treatment post-silica exposure attenuated collagen deposition and significantly reduced lung injury. These results suggest that IMP may be a potential therapy for treatment of chronic pulmonary inflammation.

Previous studies in our laboratory demonstrated that macrophage receptor with collagenous structure (MARCO), a class A scavenger receptor, is primarily responsible for silica uptake by AM [38]. However, further studies demonstrated that MARCO-null mice had increased lung inflammation and fibrosis, which was most likely due to decreased clearance of silica from the lung [39, 40]. This is why it was important in the present study to evaluate the influence of IMP on silica uptake. Because IMP did not affect silica uptake in AM, this suggests that the observed decrease in inflammation was not simply a result of altered silica uptake. In addition, it is likely that pathology would not be reversed upon termination of IMP treatment because the clearance is not affected.

Changes in lysosome membrane lipid composition may promote or reduce fusogenicity, and may stabilize or destabilize the membrane [41, 42]. A key regulator of this process is lysosomal acid-sphingomyelinase (aSMase). Inhibition of aSMase has been associated with reducing several pathological conditions such as inflammation, lung injury, and fibrosis [43–46]. It was observed in this study that IMP pretreatment significantly inhibited aSMase activity, which is consistent with existing literature [27, 28, 35, 36]. Inhibition of lysosomal aSMase activity by IMP can be a potential mechanism by which IMP exerts its anti-inflammatory effect.

When the ratio of ceramide to sphingomyelin is decreased due to aSMase deficiency, lysosome fusogenic activity is reduced [34, 42, 47]. Therefore, it was suspected that changes in vesicle membrane composition due to IMP treatment may disrupt cargo trafficking, such as the fusion of lysosomes with particle-containing phagosomes. If this phagosome maturation did not occur, then damaging lysosomal contents would not be present when the particle causes membrane permeabilization. However, experiments tracking the fluorescence of pH-sensitive *E. coli* BioParticles during phagocytosis demonstrated that IMP did not disrupt normal phagosome maturation (Additional file 1: Figure S2). Fluorescence slightly increased during the first 60 min, then decreased. Uptake of BioParticles that were incompletely quenched by trypan blue may be occurring early and BioParticles may begin to be degraded later, while phagosome maturation continues to quench fluorescence throughout. Regardless of the trends over time, it is clear that IMP did not alter BioParticle processing relative to untreated cells.

IMP significantly lowers silica-induced LMP (Additional file 1: Figure S3). This is consistent with literature suggesting sphingomyelin stabilizes membranes [48]. Also, studies show that IMP increases lysosomal cholesterol, likely by inhibiting cholesterol trafficking and sequestering it in the membrane as the lysosome expands in volume [49–52]. The phagolysosome membrane permeabilization assay (Additional file 1) utilized digitonin, which creates membrane pores by replacing cholesterol [53]. Higher cholesterol in lysosomes of IMP-treated cells causes them to be more sensitive to digitonin extraction, which would explain why IMP resulted in slightly higher cathepsin and NAG levels in cells that did not receive silica. Nonetheless, these effects were minor relative to the significant reduction in silica-induced LMP observed in cells treated with IMP.

## Conclusions

IMP reduced lung inflammation caused by short- and long-term silica exposure in a C57Bl/6 mouse model. These effects are likely a result of IMP inhibiting lysosomal acid sphingomyelinase and stabilizing the phagolysosomal membrane, while particle uptake and phagosome maturation remain unaffected. Additional studies are needed to more fully determine the mechanism of IMP action in the context of particle-induced inflammation. IMP is a U.S Food and Drug Administration approved tricyclic antidepressant drug that could be repurposed as a protective agent for lung inflammation and subsequent disease progression.

## Methods

### Mice

C57Bl/6 wild type mice were housed in a specific-pathogen-free laboratory animal facility with controlled environmental conditions and a 12 h light/dark cycle. The mice were provided with ovalbumin-free food and deionized water ad libitum. Age matched (6-8 week) male and female mice were used in all studies. All animal procedures were approved by and in accordance with the Institutional Animal Care and Use Committee at the University of Montana.

### Silica preparation

Crystalline silica (Min-U-Sil-5, Pennsylvania Glass Sand Corporation) was washed in 1 M HCl at 100 °C for 1 h followed by three washes with sterile water. Then, the silica was dried in an oven at 200 °C for several hrs to remove all water. Silica was determined endotoxin-free by Limulus Amebocyte Lysate assay (Cambrex, Walkersville, MD), data not shown. Immediately prior to each experiment, stock suspensions of silica (5 mg/ml) in phosphate-buffered saline (PBS) were dispersed by sonicating for 1 min at approximately 50% power in a 500 watt, 20 kHz cup-horn sonicator (Misonix XL-2020, Farmingdale, NY) attached to a Forma Scientific circulating water-bath at 4 °C. Scanning electron microscopy and light scattering techniques were used to determine characteristics of the silica suspensions, which has been previously published by our laboratory [54]. The zeta potential is −16.2 mV and the average particle diameter is approximately 1.5-2.0 μm; however, the particles have a relatively irregular shape and heterogeneous diameters.

### Alveolar macrophage (AM) isolation and culture

Mice were euthanized by a lethal injection of sodium pentobarbital (Euthasol, Virbac, Fort Worth, TX) and the lungs were removed with the heart. The lungs were lavaged with 1 ml of cold PBS five times. Pooled cells were centrifuged at RCF (avg) 400 x *g* for 5 min. The lavage fluid was discarded and cells were resuspended in RPMI 1640 culture media supplemented with 10% fetal bovine serum, sodium pyruvate, and an antibiotic-antimycotic solution (Mediatech, Manassas, VA). Lytic reagent (Zap-OGLOBIN II, Beckman Coulter, Brea, CA) was added to a sample of cells prior to counting with a Z2 Coulter particle counter and size analyzer (Beckman Coulter). Cells were resuspended at 1 × 10^6^ cells/ml and 0.1 ml/well was added to flat-bottom, tissue culture-treated 96-well plate (equivalent to approximately 3 × 10^5^ cells/cm^2^). Cells were incubated in a 37 °C water-jacketed CO_2_ incubator (ThermoForma, Houston, TX). In vitro and ex vivo treatments included IMP (25 μM; Sigma, St. Louis, MO), silica (50 or 100 μg/ml) and/or lipopolysaccharide (LPS, 20 ng/ml, Sigma). This IMP dose was selected because it is used for in vitro studies with various cells [55, 56] and was effective in inhibiting aSMase in alveolar macrophages (Fig. 11). The doses of silica and LPS have been shown previously in our laboratory to stimulate an inflammatory response by alveolar macrophages in vitro [39].

### In vivo treatments

Mice were exposed to 25 μl of silica in sterile PBS (1 mg/mouse) or vehicle (sterile PBS only) by oropharyngeal (OP) aspiration. Briefly, the mice were anesthetized by isoflurane inhalation and the volume was delivered into the back of the throat. By holding the tongue to the side, the solution was aspirated into the lungs. This silica concentration was selected because we have shown that it leads to inflammation and fibrosis in mice in our previous studies [31, 32, 57].

Early studies in mice often used intraperitoneal (IP) injections of IMP with doses ranging from approximately 5 to 50 mg/kg. We selected a single dose of 25 mg/kg based on more recent work relevant to our study [27, 58]. For our 24 h short-term study, mice received 100 μl of IMP (25 mg/kg IP) dissolved in sterile PBS or sham (sterile PBS alone). While IP administration is appropriate for mouse models, humans receive IMP tablets for oral administration at adult doses ranging from 50 to 300 mg/day depending on patient condition (refer to FDA label for details on specific products). These dose recommendations are for the treatment of depression; of course, additional studies are required before recommending human dosing of IMP for particle-induced inflammation.

For the six- and ten-week studies, osmotic pumps (Alzet, Cupertino, CA) were used to deliver IMP continuously over an extended period of time. Mice were anesthetized by 100 μl IP injection of a Ketamine (80 mg/kg, Putney Inc., Portland, ME) and Xylazine (12 mg/kg, AnaSed, Lloyd laboratories, Shenandoah, IA) cocktail. Skin over the implantation site was washed and shaved, then betadine was applied. An incision was made adjacent to the mid-scapular region on the back of the animal. Hemostats were then inserted into the incision and a pocket formed by opening and closing the hemostats to spread the subcutaneous tissue. The pocket was large enough to allow some free movement of the pump. The sterile pump, with PBS ± IMP, was inserted into the subcutaneous pocket with the delivery portal inserted first. The incision was then closed using vet bond (3M Animal Care Products, St Paul MN). Mice were given a dose of buprenorphine (0.05-0.10 mg/kg, subcutaneous, Reckett Benckiser Healthcare (UK) Ltd., Hull, England). Mice were monitored until they had recovered from the anesthesia.

All mice were observed daily as part of routine protocol for animal care. No overt abnormalities were reported as a result of IMP treatment.

### In vivo treatment timelines

Mice for each study were randomly designated into groups based on treatment combination (PBS ± silica; IMP ± silica). Mice in short-term studies were exposed for 24 h. Some cells isolated from short-term studies were used for additional 24 h ex vivo experiments. Mice in long-term IMP co-treatment studies were exposed to silica or vehicle weekly for the first four weeks and received IMP or sham (PBS) by osmotic pump for the entire duration (six weeks total). Mice in long-term IMP post-treatment studies were exposed to silica or vehicle weekly for the first four weeks, then received IMP or sham (PBS) by osmotic pump for the remaining duration (ten weeks total).

### Protein assays

Mouse IL-1β cytokine present in culture supernatants or lavage fluid was quantified using commercially available ELISA kits according to the manufacturer’s protocol (R&D Systems, Minneapolis, MN) with a SpectraMax 190 plate reader (Molecular Devices, Sunnyvale, CA). A standard curve was used to calculate the protein concentration as pg/ml. In vitro experiments for assessment of IL-1β involved treatment of AM ± silica (100 μg/ml) for 24 h. Total protein in the whole lung lavage fluid was measured by Pierce bicinchoninic acid (BCA) assay, according to manufacturer protocol (ThermoFisher, Rockford, IL).

### Toxicity assay

AM were treated for 24 h with silica (50 μg/ml). Cell viability was determined by MTS assay using the CellTiter^96^ assay (Promega, Madison, WI) according to the manufacturer’s protocol with one modification described below. This assay used a colorimetric dye read by a colorimetric plate reader (Molecular Devices). In order to avoid artifacts in the optical density values, steps were taken to remove the MTS reaction (transferring it into another plate) from the cell/particle mixture adhered to the plate bottom. The formation of bubbles was avoided and the plate was read at 490 nm. Data was transformed to a percent relative to the no particle, no IMP control cells.

### Histology

The lungs from each mouse were inflation-fixed through the trachea with 4% paraformaldehyde-PBS (Electron Microscopy Sciences, Hatfield, PA) and submerged in the same fixative overnight at 4 °C. The lungs were washed with cold PBS, dehydrated with ethanol, embedded in paraffin, and sectioned on a rotary microtome at 4 μm. Hematoxylin and eosin (H&E; RAS Harris Hematoxylin and Shandon Alcohol Eosin) staining was completed using a ThermoShandon automated stainer (ThermoFisher). For immunohistochemistry and picrosirius red staining, sections were placed on charged slides and dried at 56°C overnight then deparaffinized in Xylene and hydrated through descending grades of ethanol to distilled water.

During preparation for immunohistochemistry, deparaffinized slides were placed in Tris buffered saline pH 7.4 (TBS; Scytek Labs, Logan, UT) for 5 min for pH adjustment. Following TBS, slides underwent enzyme induced epitope retrieval utilizing 0.04% pepsin (Sigma) in 0.2 N hydrochloric acid at 37°C for 20 min followed by running tap water rinse as well as several changes of distilled water. Then, standard avidin-biotin complex staining steps were performed at room temperature on a DAKO Autostainer. All staining steps are followed by two rinses in TBS + Tween 20 (Scytek). After blocking for non-specific protein with Normal Rabbit Serum (Vector Labs, Burlingame, CA) for 30 min, sections were incubated with avidin-biotin blocking system for 15 min each (Avidin D, Vector Labs; d-Biotin, Sigma). Primary antibody slides were incubated for 60 min with the monoclonal rat anti-mouse Ly-6B.2 neutrophil marker (clone 7/4; AbD Serotec, Raleigh, NC) diluted 1:2500 in Normal Antibody Diluent (NAD; Scytek). Biotinylated rabbit anti-rat IgG (H + L), mouse absorbed, at 10 μg/ml in NAD was incubated with the slide for 40 min followed by Alkaline Phosphatase Reagent (Kirkegaard & Perry Laboratories, Gaithersburg, MD) for 60 min. Reaction development utilized Vector Substrate Kit 1 (Fast Red) phosphatase chromogen for 8 min. This was followed by counterstain in Gill 2 Hematoxylin (ThermoFisher) for 15 s, differentiation, dehydration, clearing, and mounting with Permount mounting media.

Deparaffinized slides designated for picrosirius red were first stained for nuclei using Weigerts Hematoxylin for 10 min followed by distilled and running tap water rinses. Slides were incubated in 0.2% aqueous Phosphomolybdic acid for 2 min, washed in running tap and distilled water, then stained with 0.1% aqueous Picrosirius Red for 90 min. Stain was followed by several changes of distilled water, dehydration, clearing, and mounting.

Semi-quantitative multiparametric analysis of H&E-stained lung tissue sections was completed using an ordinal scale ranging from no significant pathology (score: 0) to severe pathology (score: 5). Specifically, severity categories were classified according to percentage of affected lung tissue section: less than 10% (minimal: 1), 10-25% (mild: 2), 25-50% (moderate: 3), 50-75% (marked: 4), and greater than 75% (severe: 5). Parameters were alveolar proteinosis, alveolitis (includes mixed inflammatory cell infiltrates, microgranulomas, and associated interstitial fibrosis), perivascular/peribronchiolar lymphoid aggregates, and type II alveolar epithelial cell hyperplasia.

A board-certified veterinary pathologist with expertise in respiratory pathobiology of inhaled toxicants in laboratory rodents examined the prepared lung tissue sections by light microscopy for qualitative and semi-quantitative analysis. Tissues were examined from at least three mice per treatment group, with at least three tissue sections per mouse for scoring. The pathologist had no knowledge of the exposure history of the individual mice prior to his assessment of pulmonary histopathology.

### Hydroxyproline assay

Hydroxyproline is an amino acid that is a major component of collagen. The hydroxyproline assay was used to quantify collagen content, as previously described [59]. Lung lobes were excised, weighed, and immediately frozen. The lung tissues were homogenized using a tissue homogenizer (Tissue Tearor model 985,370, Biospec Products, Bartlesville, OK), in 1 ml of sterile PBS. An aliquot of lung homogenate was hydrolyzed in 12 N HCl at 110 °C for 24 h. The mixture was reacted with chloramine T and Ehrlich’s reagent to produce a hydroxyproline-chromophore that was quantified by spectrophotometry at 550 nm. Hydroxyproline content was determined by triplicate analysis of the samples to provide an average value.

### Particle uptake

Silica particle internalization was determined by flow cytometry using a side-scatter technique previously described [38]. Isolated AM were cultured as described above ± IMP and ± silica (50 μg/ml) for 90 min in suspension cultures using 1.5 mL microfuge tubes and end over end tumbling (Lab Quaker Shaker, ThermoForma). The cells were centrifuged and washed once in PBS. The resulting cell pellet was resuspended in PBS and transferred to filter-top flow cytometry tubes (BD Biosciences, San Jose, CA) for analysis. Data was expressed as mean side scatter for 10^4^ cells.

### Acid sphingomyelinase assay

Isolated AM were treated ± IMP (25 μM) for 30 min, then exposed to LPS (20 ng/ml) only, or silica (100 μg/ml) and LPS for 2 h. Cell lysates were prepared from the AM using Dounce homogenizer (tight pestle). Cell lysates were assayed for acid sphingomyelinase with commercially available kits according to the manufacturer’s protocol (K-3200 Echelon Biosciences Inc., Salt Lake City, UT). Fluorescence analysis was done using a Gemini XS plate reader (Molecular Devices) at 360 nm ex and 460 nm em. Data were expressed as pmol/h of active acid sphingomyelinase.

### Statistical analysis

Semi-quantitative analysis of histopathology followed scoring standards that indicate use of an ordinal rating scale, which violates assumptions of normality required by parametric statistical methods [60]. Therefore, unweighted relative effects hypotheses in this two-way factorial design were tested by calculating the ANOVA-type statistic (ATS) [61, 62] followed by nonparametric multiple comparisons by Dunn’s test with *p*-values adjusted in contrasts of interest using the Holm-Bonferroni method. All other statistical analyses involved comparison of means using a two-way ANOVA followed by multiple comparisons using Tukey’s honest significant difference (HSD) test to compensate for increased type I error. Statistical significance was defined as a probability of type I error occurring at less than 5%, and significant contrasts of predetermined interest are shown as *p < 0.05, **p < 0.01, and ***p < 0.001. Data is represented as the mean ± standard error of at least three independent replications for each experiment. White bars indicate no IMP treatment and gray bars indicate IMP treatment. Graphics and analyses were performed on GraphPad Prism and R statistical software, respectively.

## Additional files

Additional file 1: Supplementary methods, figures, and references. **Figure S1.** In vitro IL-1β release from THP-1 cells treated with silica ± imipramine. **Figure S2.** Assessment of phagosome maturation using fluorescein-conjugated *E. coli* BioParticles. **Figure S3.** Effect of imipramine on silica-induced phagolysosome permeabilization. (PDF 4645 kb)
