# Supplementary material for: Imipramine blocks acute silicosis in a mouse model
Source: Part Fibre Toxicol. 2017 Sep 11;14:36. doi: 10.1186/s12989-017-0217-1 (PMC5594487; doi:10.1186/s12989-017-0217-1)
Supplement: Supplementary file 1 — Supplementary methods, figures, and references. Figure S1. In vitro IL-1β release from THP-1 cells treated with silica ± imipramine. Figure S2. Assessment of phagosome maturation using fluorescein-conjugated E. coli BioParticles. Figure S3. Effect of imipramine on silica-induced phagolysosome permeabilization. (PDF 4645 kb) [file 12989_2017_217_MOESM1_ESM.pdf]

Additional File 1 as follows is a supplement to: Biswas R, Trout KL, Jessop F, Harkema JR, Holian A. Imipramine blocks acute silicosis in a mouse model. Part Fibre Toxicol.

Supplementary Methods

THP-1 cell culture

The human THP-1 acute monocytic leukemia cell line was cultured according to supplier (ATCC) recommendations in RPMI 1640 media with 10% fetal bovine serum and an antibiotic/antimycotic solution. THP-1 cells in suspension were differentiated into macrophage-like cells by adding 1,25-dihydroxyvitamin D3 (150 nM) for 24 hours, as previously described by our laboratory [29]. Then, groups of cells were treated with IMP (25 µM) for 30 min. Finally, all cells received phorbol 12-myristate 13-acetate (PMA; 5 nM), all cells received lipopolysaccharide (LPS; 10 ng/ml), and groups of cells received silica (50 µg/ml). Supernatants were collected after 24 hrs for analysis of IL-1β by ELISA.

Bone marrow-derived macrophage culture

C57Bl/6 mice were euthanized by lethal injection of sodium pentobarbital, then their hind legs were removed. Bone marrow from the femur and tibia was flushed and suspended in RPMI 1640 culture media supplemented with 10% fetal bovine serum, sodium pyruvate, and a penicillin-streptomycin solution. Cells were added to flasks at 4-6x10^5 cells/cm^2 and cultured for one day at 37°C in a water-jacketed CO_2 incubator. On day 2, adherent stromal cells were discarded and suspended cells were added to flasks at 2-3x10^5 cells/cm2 with 20 ng/ml macrophage colony-stimulating factor (M-CSF; R&D Systems). Cells received additional 10 ng/ml M-CSF on days 4 or 5. Half of the media volume was replaced with fresh media and 10 ng/ml M-CSF was added on days 7 or 8. The bone marrow-derived macrophages (BMdM) are collected by trypsin and scraping for use with experiments anytime between days 10-12. Our laboratory group has shown previously that this culture method results in macrophages that express CD11b and F4/80, as measured by flow cytometry [59].

Phagosome-Lysosome Fusion Assay

Escherichia coli K-12 strain BioParticles™ conjugated with fluorescein (Thermo Fisher Scientific, Waltham, MA) were reconstituted at 20 mg/ml in sterile PBS with 2 mM sodium azide. A homogeneous suspension was prepared by vortex mixing twice for 15 sec and gentle sonication for 60 sec in a Branson Ultrasonic Cleaner 1510R-DTH at 4°C with output of 70 watt at 42 kHz ± 6%. BioParticles in stock solution were counted using a hemacytometer.

Solutions of various pH ranging from 5.00 to 7.40 were prepared by adding HCl to PBS. Then, 5 µl of BioParticle stock solution was added to 495 µl of each pH-set solution, resulting in a concentration of 2.2x10^8 BioParticles/ml. Fluorescence at 494/521 nm (ex/em) was measured immediately and at multiple other timepoints throughout a 120 min period using a Molecular Devices SpectraMax M4 plate reader with samples in a black-walled, black-bottomed 96-well plate.

BMdM cells in suspension were treated with 25 µM imipramine (IMP), 100 nM bafilomycin A1 (BAF), or remained untreated. After incubating for 30 min with gentle rotation in 37°C incubator, BioParticles were added at 1:100 Multiplicity of Infection (MOI). BMdM were incubated with BioParticles for 15 min to allow phagocytosis to occur. Then, trypan blue was added at a 1:10 ratio (resultant concentration 0.36 mg/ml) for 2 min to quench fluorescence of remaining extracellular BioParticles that were not phagocytosed. Cells were washed and analyzed with an Attune NxT flow cytometer (Thermo Fisher Scientific) as soon as possible. Cells were incubated at 37°C with gentle rotation between timepoints. Samples of cells were

placed on a glass slide for imaging with a Zeiss AxioSkop fluorescent microscope approximately 120 min after quenching non-phagocytosed BioParticles.

Phagolysosome Membrane Permeability Assay

Phagolysosome membrane permeabilization was assessed as previously described by our laboratory [57] using methods modified from Aits et al [53]. Reagents for this assay include three specialized buffers. Digitonin extraction buffer: 250 mM sucrose, 20 mM HEPES, 10 mM KCl, 1.5 mM $MgCl_2$, 1 mM EDTA, 1 mM EGTA, pH 7.5 with fresh 0.5 mM pefabloc (Sigma-Aldrich) and digitonin (Sigma-Aldrich). Cathepsin reaction buffer: 50 mM sodium acetate, 4 mM EDTA, pH 6.0 with fresh 0.5 mM pefabloc, 8 mM DTT, and 50 µM cathepsin substrate (Z-Phe-Arg-AFC; EnzoLife Sciences). N-acetyl-β-D-glucosaminidase (NAG) reaction buffer: 0.2 M sodium citrate, pH 4.5 with 300 µg/mL 4-methylumbelliferyl NAG (Sigma-Aldrich). Fluorescence of the cathepsin and NAG reaction buffers was measured using a Molecular Devices SpectraMax M4 plate reader at 400/489 and 356/444 nm (ex/em), respectively.

BMdM were plated at 2 x 10^5 cells/well in a 24-well plate, incubated at 37°C overnight, and then exposed to vehicle (PBS), silica (100 µg/mL), and/or imipramine (25 µM) for 4 hrs. Next, BMdM were washed twice with PBS and incubated with 200 µL of digitonin extraction buffer for 15 min on ice with rocking. Digitonin was titrated to determine the optimal concentration (10 µg/mL) that causes permeabilization of the plasma membrane while leaving the lysosomal membranes intact, allowing for extraction of the cytosolic fraction. Cytosolic cathepsin activity was measured 25 min after adding 50 µL cytosolic fraction to 50 µL cathepsin reaction buffer. Cytosolic NAG activity was measured 20 min after adding 30 µL cytosolic fraction to 100 µL NAG reaction buffer. LDH activity was measured following manufacturer instructions (CytoTox 96; Promega, Madison, WI). Extracted cytosolic LDH activity was used as an internal control to which cytosolic cathepsin or NAG activities were normalized. Cytosolic extract enzyme activities were calculated as a percent of total lysate activity in which a high concentration (200 µg/mL) of digitonin was used to completely lyse both the cell membrane and lysosomes.

References

29. Hamilton RF, Buckingham S, Holian A. The effect of size on Ag nanosphere toxicity in macrophage cell models and lung epithelial cell lines is dependent on particle dissolution. Int J Mol Sci. 2014;15:6815-30.
53. Aits S, Jaattela M, Nylandsted J. Methods for the quantification of lysosomal membrane permeabilization: a hallmark of lysosomal cell death. Methods Cell Biol. 2015;126:261-85.
57. Jessop F, Hamilton RF, Jr., Rhoderick JF, Fletcher P, Holian A. Phagolysosome acidification is required for silica and engineered nanoparticle-induced lysosome membrane permeabilization and resultant NLRP3 inflammasome activity. Toxicol Appl Pharmacol. 2017;318:58-68.
59. Migliaccio CT, Buford MC, Jessop F, Holian A. The IL-4Ralpha pathway in macrophages and its potential role in silica-induced pulmonary fibrosis. J Leukoc Biol. 2008;83:630-9.

Supplementary Figures

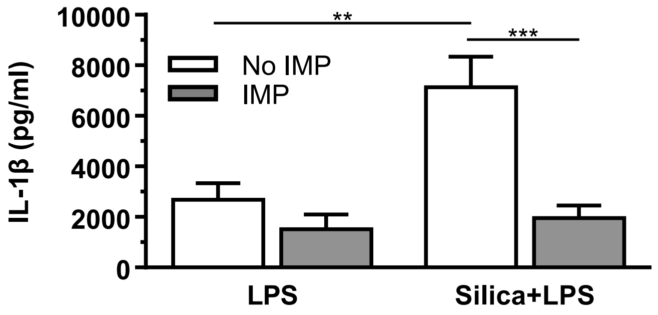

Fig. S1: *In vitro* IL-1β release from THP-1 cells treated with silica ± imipramine. THP-1 treatment conditions include IMP for 30 min, followed by exposure to PMA, LPS, and silica for 24 hrs. IL-1β release was measured by ELISA. n≥3 independent replicates per treatment condition.

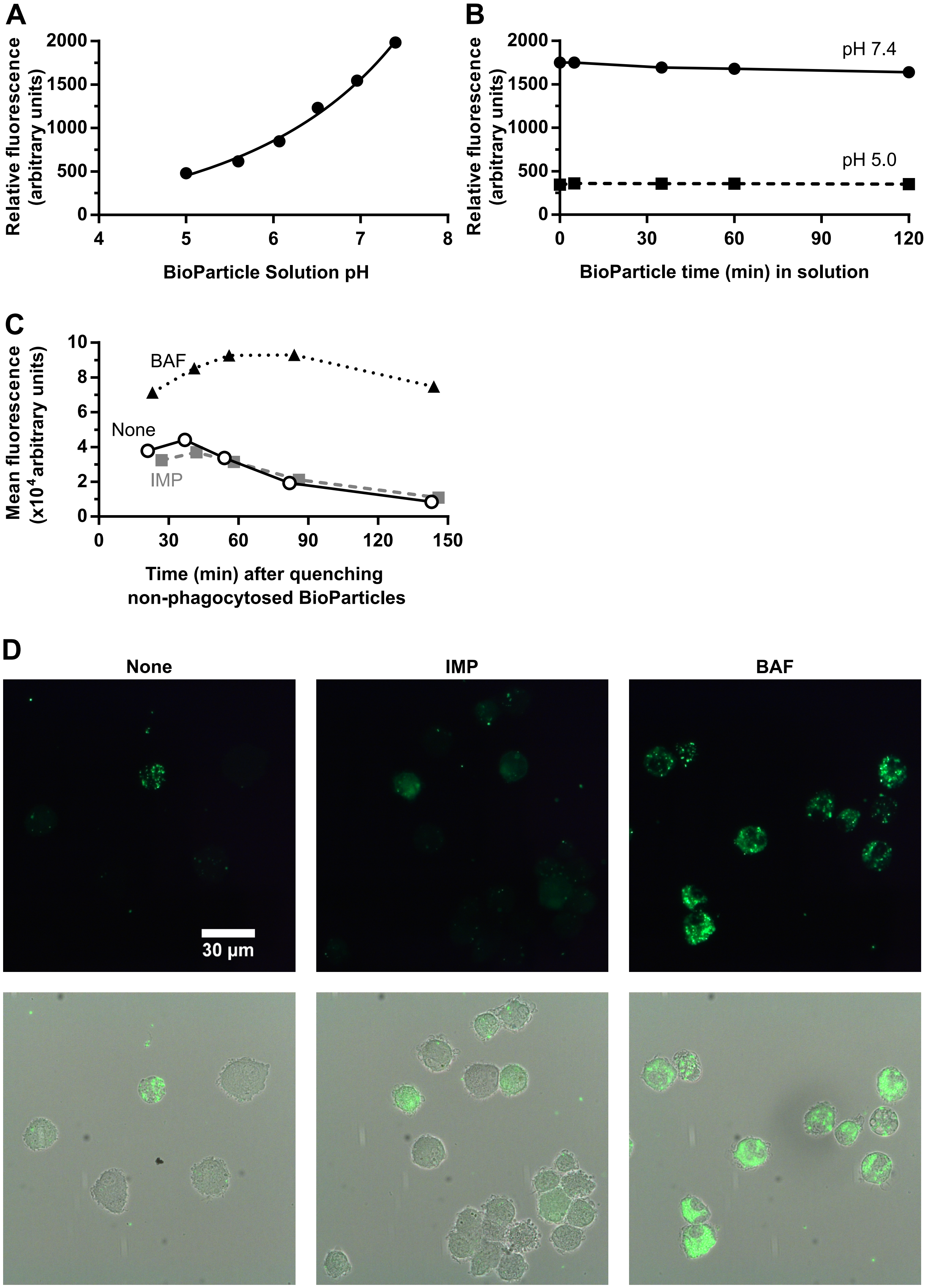

A
Relative fluorescence (arbitrary units)
2000
1500
1000
500
0
4
5
6
7
8
BioParticle Solution pH
B
pH 7.4
pH 5.0
0
30
60
90
120
BioParticle time (min) in solution
C
Mean fluorescence ($x10^4$ arbitrary units)
10
8
6
4
2
0
BAF
None
IMP
0
30
60
90
120
150
Time (min) after quenching non-phagocytosed BioParticles
D
None
IMP
BAF
30 µm

Fig. S2: Assessment of phagosome maturation using fluorescein-conjugated *E. coli* BioParticles. BioParticles were added to acellular solutions with a range of pH. Fluorescence is quenched at an acidic pH **(A)** and this quenching occurs immediately **(B)**. BMdM cells were treated with IMP, BAF or remained untreated for 30 min prior to addition of BioParticles. BioParticle fluorescence is quenched as phagosomes mature by fusing with acidic lysosomes in IMP and untreated cells, while fluorescence intensity remains high in control cells treated with BAF, an inhibitor of lysosomal acidification **(C)**. Differences in BioParticle fluorescence intensity can also be observed in representative microscope images acquired approximately 135 min after quenching non-phagocytosed BioParticles **(D)**; bottom row contains images merged with brightfield.

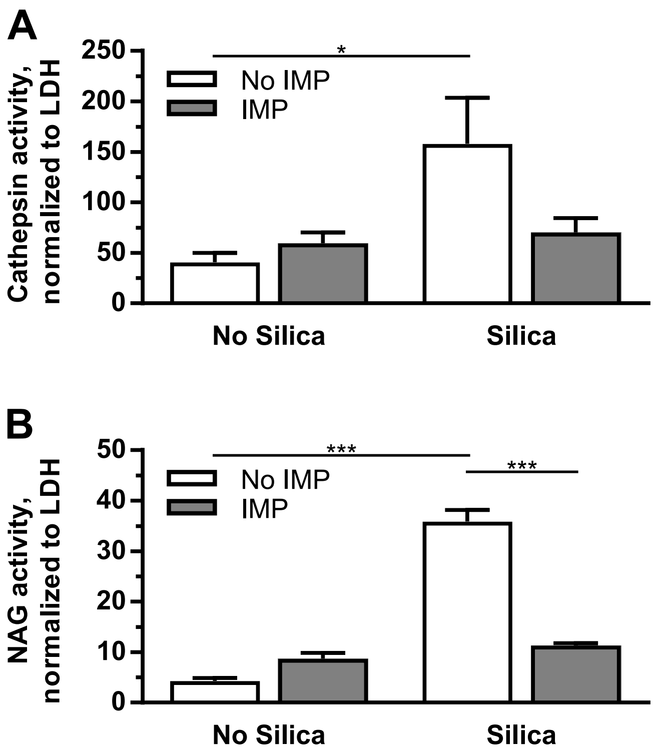

Fig. S3: Effect of imipramine on silica-induced phagolysosome permeabilization. The cytosolic fractions of BMdM treated ± silica and ± IMP were isolated to measure indicators of phagolysosome permeabilization: cathepsin **(A)** and NAG **(B)**, relative to total and normalized to LDH. n≥3 mice per treatment condition.
